# Movement Coordination during Conversation

**DOI:** 10.1371/journal.pone.0105036

**Published:** 2014-08-13

**Authors:** Nida Latif, Adriano V. Barbosa, Eric Vatiokiotis-Bateson, Monica S. Castelhano, K. G. Munhall

**Affiliations:** 1 Department of Psychology, Queen’s University, Kingston, Ontario, Canada; 2 Department of Electronics, Universidade Federal de Minas Gerais, Belo Horizonte, Minas Gerais, Brazil; 3 Department of Linguistics, University of British Columbia, Vancouver, British Columbia, Canada; 4 Department of Otolaryngology, Queen’s University, Kingston, Ontario, Canada; The University of Chicago, United States of America

## Abstract

Behavioral coordination and synchrony contribute to a common biological mechanism that maintains communication, cooperation and bonding within many social species, such as primates and birds. Similarly, human language and social systems may also be attuned to coordination to facilitate communication and the formation of relationships. Gross similarities in movement patterns and convergence in the acoustic properties of speech have already been demonstrated between interacting individuals. In the present studies, we investigated how coordinated movements contribute to observers’ perception of affiliation (friends vs. strangers) between two conversing individuals. We used novel computational methods to quantify motor coordination and demonstrated that individuals familiar with each other coordinated their movements more frequently. Observers used coordination to judge affiliation between conversing pairs but only when the perceptual stimuli were restricted to head and face regions. These results suggest that observed movement coordination in humans might contribute to perceptual decisions based on availability of information to perceivers.

## Introduction

Human conversation, like all animal communication, is a cooperative activity and those involved naturally coordinate their sensory and motor behavior. This phenomenon of human interpersonal coordination has been well established: Studies have demonstrated that interacting individuals unintentionally synchronize their nonverbal and linguistic behavior along many levels of social interaction [1]–[3]. Talkers in conversation spontaneously assimilate facial expressions, postures, pronunciation and speech rates [4]–[6]. Coordination persists even when individuals are instructed not to synchronize [7]. The seemingly automatic nature of coordination may partly result from inherent biological and behavioral rhythms and a coupling of the conversation-engaged individuals’ perceptions of each other [3], [8]. During conversation, talkers perceive others’ behavior and in turn, react or guide their behavior in an anticipatory or predictive manner [9].

Existing studies on coordinated behavior have been conducted in a number of social situations. Correlations between rated behavior and socio-cognitive variables have been demonstrated in instances of clinician-patient therapy success, rapport building and cooperative activities [10]–[13]. Group identity and familiarity have an impact on the magnitude of observed coordination. Familiarity cues such as proximity between talkers, number of gestures, eye contact and postural similarity have been examined [14], however, their contributions to our perceptions of others are still unclear. Some studies have shown that individuals identifying with others based on like-mindedness and pairs of friends are perceived to coordinate more in nonverbal and linguistic properties than those unfamiliar to them or their preferences [15]–[16].

Coordination may help establish and subsequently indicate rapport and familiarity during social interaction [10], [15]. Presenting and recognizing coordination is an important underlying process that social animals, in general, use to maintain communication and relationships. Cross-species evidence reveals that animals such as horses, dolphins and monkeys form friendships (social bonds unrelated to mating) resulting in convergent, cooperative behavior (e.g. Chimpanzees coordinate affiliative facial expressions and dolphins synchronize surfacing to indicate group identity [17]–[18]). Not only do these species form enduring bonds but they also learn to recognize those bonds in others [19]. Likewise, humans have also demonstrated remarkable social recognition accuracy in a variety of domains such as personality, social status and mental state [20]. Monitoring others’ intentions and actions accurately in this manner is a prerequisite for modulating and guiding our own behavior, interactions, and successful relationship formation [21]. The ability to use interpersonal cues to modulate behavior is impaired in many social and psychological disorders such as autism spectrum disorder and schizophrenia, leading to difficulty in successful interaction [22].

Although coordination and synchrony have been extensively documented, methods used to conduct these investigations vary drastically. Much research in this area uses measurements of behaviors that are not directly related to conversation; for example, synchronizing a pendulum swing while conversing [23] or using movement coding by independent observers to quantify coordination [24]. Further, many studies address interpersonal coordination by equating it to mutual entrainment, often analyzed by quantifying behavioral recurrence. Entrainment refers to the phase-locked and temporally stationary process associated with coordination (e.g. walking in step with another individual) [25] or postural sway generally resulting from inherent biological processes such as respiration [7], [26]. Temporally stables, or quasi-stable, behaviors undoubtedly contribute to conversational rhythms, but there are many other potential sources of movement similarity whose effects on production and/or perception need to be explored. For example, examining how interpersonal coordination affects observers’ perception of others is still unexplored although it has been weakly identified as a cue in determining familiarity [14].

In our studies, we used novel computational methods to quantify *time-varying* motor correspondence between conversing individuals. We demonstrated that observed coordination between conversing individuals (interlocutors) varied based on the affiliation (i.e. familiarity) between the talkers (i.e. whether they were friends or strangers). Further, we explored how these coordination cues may contribute to external observers’ perceptual judgments about the relationship between interlocutors.

## Experiment 1: Movement Analysis

This experiment investigated whether movements of interacting talkers are more highly coordinated than between non-interacting (i.e. randomly paired) participants. More specifically, we investigated whether there was an observable variation in coordination between individuals as a result of known affiliations. We quantified motoric coordination using Correlation Map Analysis (CMA) to motion signals from video [27]. Although automated movement analysis is not new in the measurement of social coordination (e.g., [28]), the approach we take has two main advantages over other existing techniques. First, we use the Horn and Schunck [29] algorithm for computing optical flow (Note: Optical flow refers to the pattern of motion of objects, surfaces, textures and edges based on relative motion between the observer and the scene. This concept of optical flow allows us to understand how a visual scene is perceived by animals in a moving world in order to discern the possibility of action within an environment. The Horn & Schunck method is a standard method with which optical flow has been computationally conceptualized in vision research) to assess talker movement; this is a more sensitive measure of motion in video than other techniques such as frame-differencing [28]. Second, the assessment of coordination uses CMA, a filtering technique (see Methods) well-suited for non-stationary time series, to measure behavioral correspondences that that in natural communication are rarely strictly cyclical. In addition, the approach taken here provides us with a more practical approach to initially examine how observers may perceive coordinated movement as it presents a more holistic treatment of the motion components rather than focusing on more specific behaviors (e.g. postural sway). Research in spoken language convergence suggests that observers perceive acoustic similarity using multiple acoustic cues [6] and not all cues are present all of the time. We believe that observers may use multiple movement cues which can be captured by our approach.

### Methods

#### Participants

Sixty-two undergraduates (Mean Age = 21.2; 36 females) participated in this experiment (The General Research Ethics Board (GREB), by means of a delegated board review has cleared the proposal for the study entitled “GPSYC-612013 The contribution of linguistic and behavioural cues in making social judgments” for ethical compliance with the Tri-Council Guidelines (TCPS) and Queen’s Ethics policies). Twenty-seven same-gender dyads and 4 mixed-gender dyads were recruited as friend pairs or were experimentally paired up to form stranger pairs. All pairs completed a questionnaire indicating the length of their relationship in months and its quality (e.g. ‘How well do you know your conversation partner?’) on a scale from 1 (Not at all) to 7 (Extremely well). All strangers were verified to be unacquainted.

#### Stimuli

Dyads engaged in unstructured conversation for approximately 10 minutes without the experimenter present to ensure the most natural conversation possible. Participants were asked to engage in conversation regarding any topic and in any language they felt most comfortable with. They were also provided with a list of suggested topics to rely on if needed, however, no participants in this experiment referred to this list or spoke in a language other than English. All conversations were recorded using a high-definition video camera (Sony HD Handycam, Model HDR-XR550) while participants sat in fixed chairs approximately 35 cm apart. The audio component from each video was removed and 31 videos were analyzed.

Optical flow analysis was used to quantify the motion associated with specific regions of interest (ROI), such as a participant’s head, for each video. Optical flow is a standard computer-vision technique for extracting 2D measures from video. In this technique, consecutive frames of the video are compared for changes of pixel intensity. The amount and direction of motion associated with each pixel from one frame to the next is inferred from the intensities of neighboring pixels [29]. Over a sequence of images, this results in a series of velocity vectors consisting of amplitude and 2D direction. For our purposes, we discard the directional information and use only the amplitudes, summing them for all pixels within the user-defined ROI for each frame step [27]. The resulting time series represents the total motion in an identified region.

**Figure 1:**
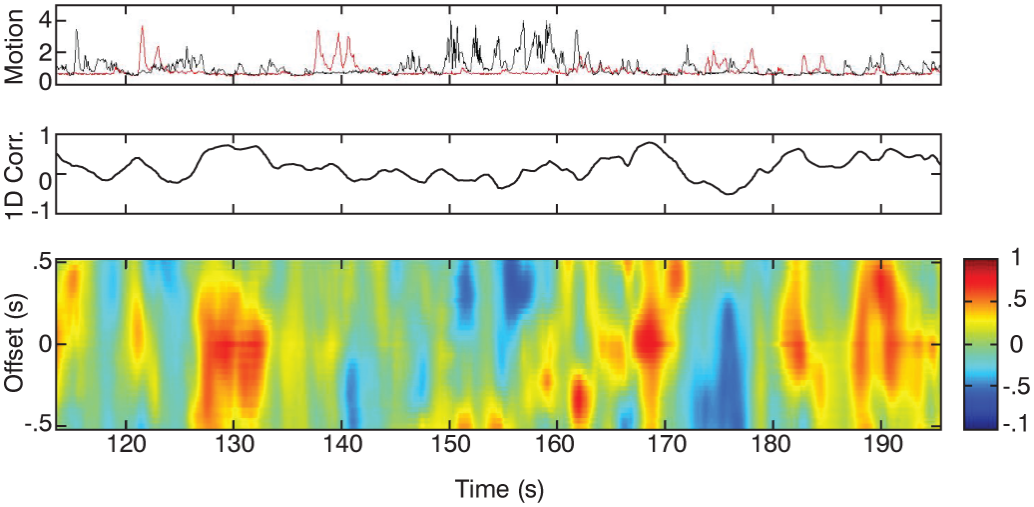
Correlation map Analysis. This method was used to compute time-varying coordination between interlocutors. This method computes the correlation between a pair of signals as a function of both time and delay between the signals resulting in a 2D correlations map (Barbosa et al, 2012). Here, the top panel indicates the motion of the two individuals in red and black. The second panel shows instantaneous correlation with no time lag introduced (lag 0). The third panel shows that some coordination can only be captured by introducing a lag between the conversing individuals’ motion. The red color indicating higher correlation demonstrates how conversations change and evolve in the amount of coordination present.

CMA is then used to compute coordination between pairs of motion signals corresponding to the identified regions of interest such as the heads of each conversant. Using a bi-directional moving window filter, velocity difference values from a small ‘window’ of frames from one signal are compared to the second. The instantaneous correlation between the two signals is computed for every possible offset within +/−0.5 s (Note: 0.5 s was selected based on evidence regarding the timing and duration of “conversational events” indicating that individuals engaged in conversations are able to predict movements and speech of those they are speaking within 500 ms [30]–[32]. With the assumption that coordinated events follow similar timelines, this value was selected to capture relevant information. In other words, a correlation value is produced from each point in time based on both preceding and following values in the time-series. The bi-directional time lag allows for alternating behavior such as that seen in social interaction, to be quantified in terms of time-varying coordination [27], [33]. The value of introducing these time lags when analyzing conversation motion is demonstrated in Figure 1 where the amount of motion changes and the evolution of conversational correlation can be observed.

As we have applied the technique here, CMA was computed on the total motion produced by our talkers at any point in time (the summation of the optical flow measure). The “instantaneous” correlation is a linear estimate of the covariance of the two talkers’ motion defined over a very short temporal span (exponentially decaying weights forward and backward in time). The correlation, thus, assesses changes in amount of motion over small windows. It does not require that the movements be equal in size but that the relative amount of motion change be linearly related. This approach has many advantages. It does not make any assumptions about the form of the conversational convergence. The measure does not treat movement by the same effectors or in the same Cartesian or joint-centered coordinates as special yet these actions will be measured and assessed by their global spatio-temporal structure. Further, it does not require that the coordination between talkers be constant for long periods of time such as a measure of entrainment would; rather, it is sensitive to momentary fluctuations in how talkers interact.

In this study, CMA was applied to broad regions of interest (ROIs) identified around each talker (to encompass all talker motion including arm motion). However, CMA could be used with any two movement signals. Here, the global motion for each interlocutor was correlated with their partner. Higher correlation values indicated high coordination between the interlocutors, in general. In the future, specific articulator coordination could be tested using the same technique.

Average distributions of correlation for the friend and stranger pairs were created. These distributions were simplified to look at only positive lags (i.e., from synchronous (0) to an offset of +0.5 s), resulting in 16 lags in total, including 0 (Frame rate = 30 fps, 0.5 s = 15 frames plus one 0 lag frame) (Note: Although only positive lags were used, analysis of all lags produced the same results). For statistical comparison, a permutation sampling approach [34] was used to create a null distribution. Motion from all possible pairs with the exception of talkers correlated with themselves and true pairs was correlated (The motion estimates computed from the optical flow data from each ROI around each talker are stored as separate signals. This allows for motion from one individual to be correlated with motion from any other individual in the data set). Order of talker designation was not important so redundant pairs were eliminated resulting in a null distribution of 1860 pseudo-pairs. The probability of the mean correlation at each lag for both friends and strangers was estimated based on a permutation test using the null distribution to determine whether real conversations produced more extreme correlations than chance.

To observe correlation differences between categories of affiliation more effectively, the friend and stranger distributions were subtracted from one another to create a friends-strangers difference distribution. Comparisons were made based on two different null difference distributions generated using the resampling method: 1) The real-pair null distribution was created by assigning 24 real pairs (12 friend and 12 stranger pairs) randomly to two arbitrary groups and computing the difference between the groups. This was repeated for 1000 iterations and the average of the differences was used for the final real-pair null difference distribution. This distribution was used to determine differences between data sets containing real conversational motion but arbitrary affiliation designation. 2) The random-pair null distribution was created by making 24 random pairings with 48 individuals. Real-pair combinations were eliminated. Twenty-four pseudo-pairs were randomly assigned to two groups and the difference was computed. As with the real-pair null distribution, the pairing and differencing process was computed for 1000 iterations and the average of these differences was used for the random-pair null difference distribution.

All distributions were normalized to represent proportional differences.

### Results

**Figure 2:**
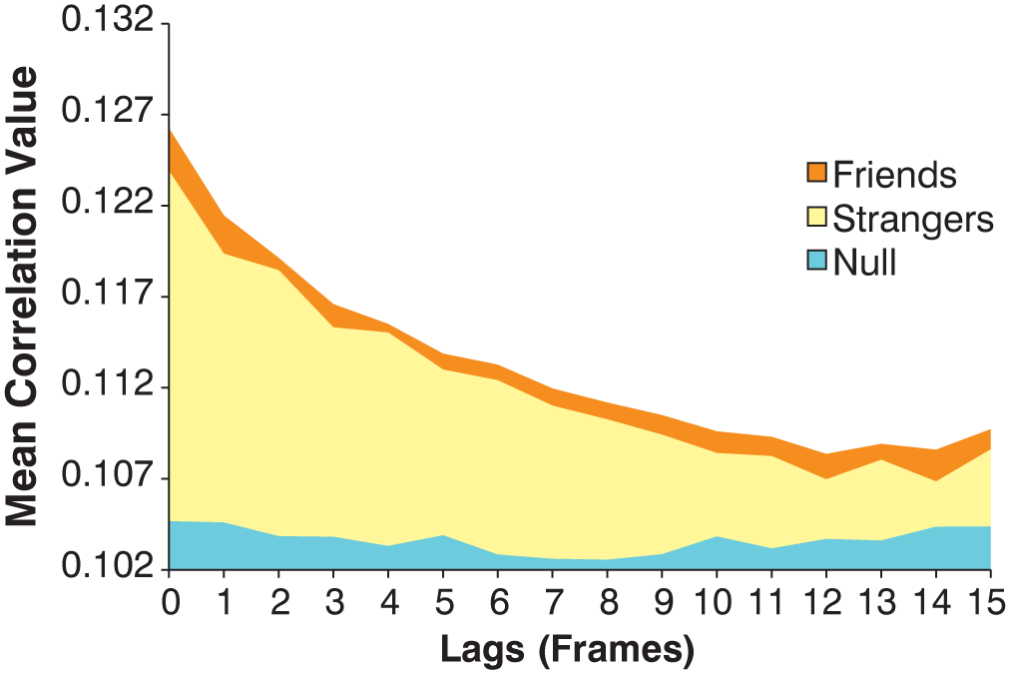
Mean correlation value for friends and strangers compared to a null distribution. Individuals in conversation, in general, shows significantly higher correlations than randomly paired motion, especially in the earlier lags closes to synchronous. Friends show significantly higher correlations than strangers at all lags.

Our analyses demonstrated that conversations between both friend and stranger pairs resulted in significantly higher correlation than randomly paired motion (p<0.05) (Figure 2). This was especially apparent in lags closest to synchronous. These results indicate that individuals engaged in conversation are highly sensitive to their partners’ movements and the correlation at synchrony suggests that interlocutors may be predicting each others’ behavior [35]. Standard simple reaction times for voice and eye movements [36], [37] are approximately 200 ms and for more complex decisions are much greater. Thus, listeners appear to be using ongoing movement information to anticipate the other talker.

**Figure 3:**
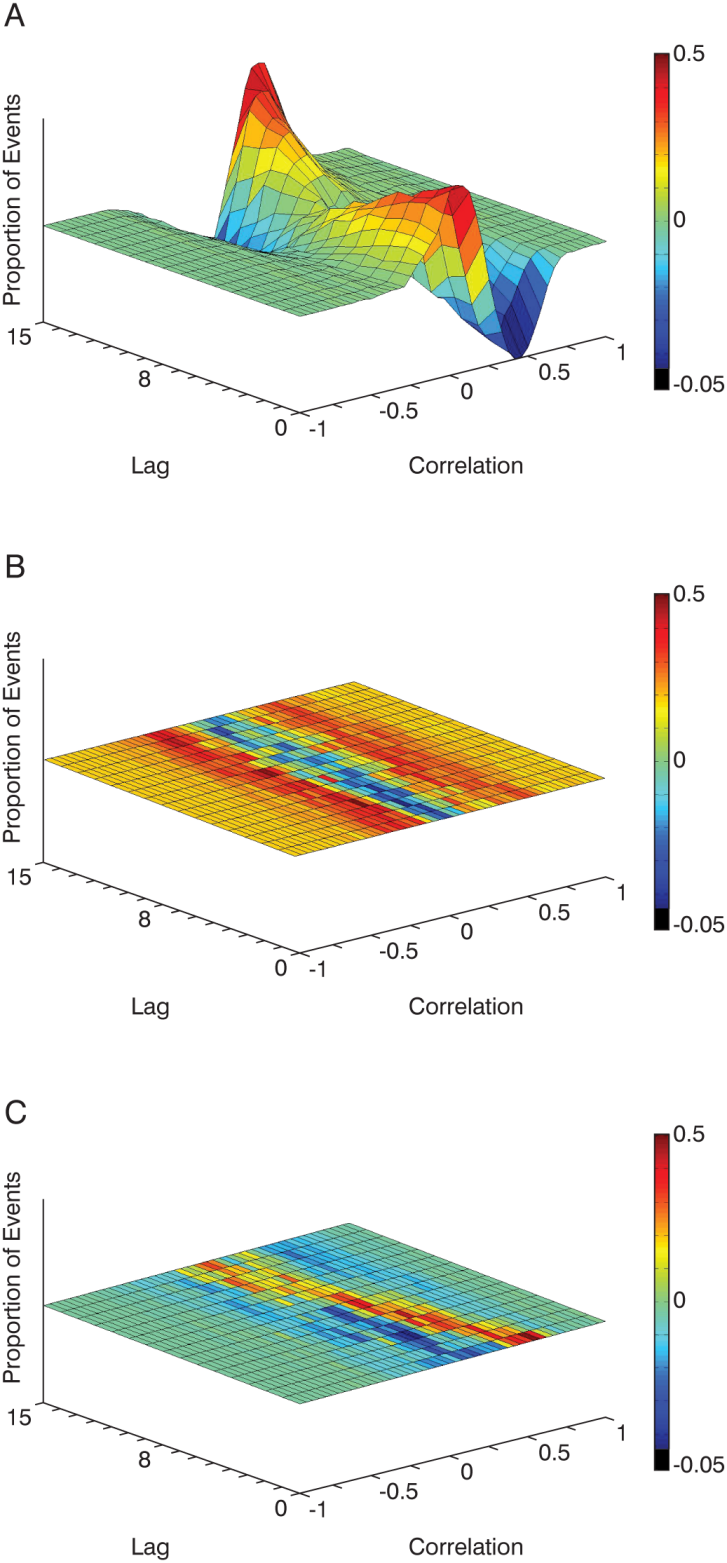
Three-dimensional average correlation difference distributions for A) friends-strangers, B) random-pair subtractions and C) real-pair random subtractions. Redder colors indicate higher correlation differences along the x-axis with height indicating frequency of events. Lag counts (in frames) indicate 16 temporal points between 0 and 0.5 s where average correlation was computed. Greater positive peaks indicate more correlated events for friends in comparison to strangers.

Mean correlation differences between true friends and strangers at all lags were significantly greater than both null difference distributions where friends had higher correlation than strangers (p<0.0001) (Figure 3). These comparisons suggest that friends’ and strangers’ conversations contain coordination content unique to their affiliation categorization. This was confirmed by comparison to both randomly paired motion (random-pair null difference distribution) and true conversational motion (real-pair null difference distribution).

Experiment 1 demonstrated in a quantified manner that correlation was an inherent part of conversational movement and that friends coordinated more than strangers. This supported previous studies that indicate that familiarity and good rapport result in linguistic and behavioral coordination [11], [15]. The correlation data from this experiment were used to identify stimuli for perceptual judgments in Experiments 2 and 3.

## Experiment 2 and 3: Perceptual Judgment Task

In Experiments 2 and 3, we investigated whether observers were perceptually attuned to the correlational structure identified in Experiment 1 and whether perception of coordination differences affected the accuracy of affiliation judgments. Specifically, we isolated sources of information (head movement coordination, full body coordination and only body coordination) to explore how observers may be influenced by the availability of perceptual cues. Experiment 3 was conducted to control for differences in correlation between the full-body movement and head movement in Experiment 2.

### Method

#### Stimuli

**Figure 4:**
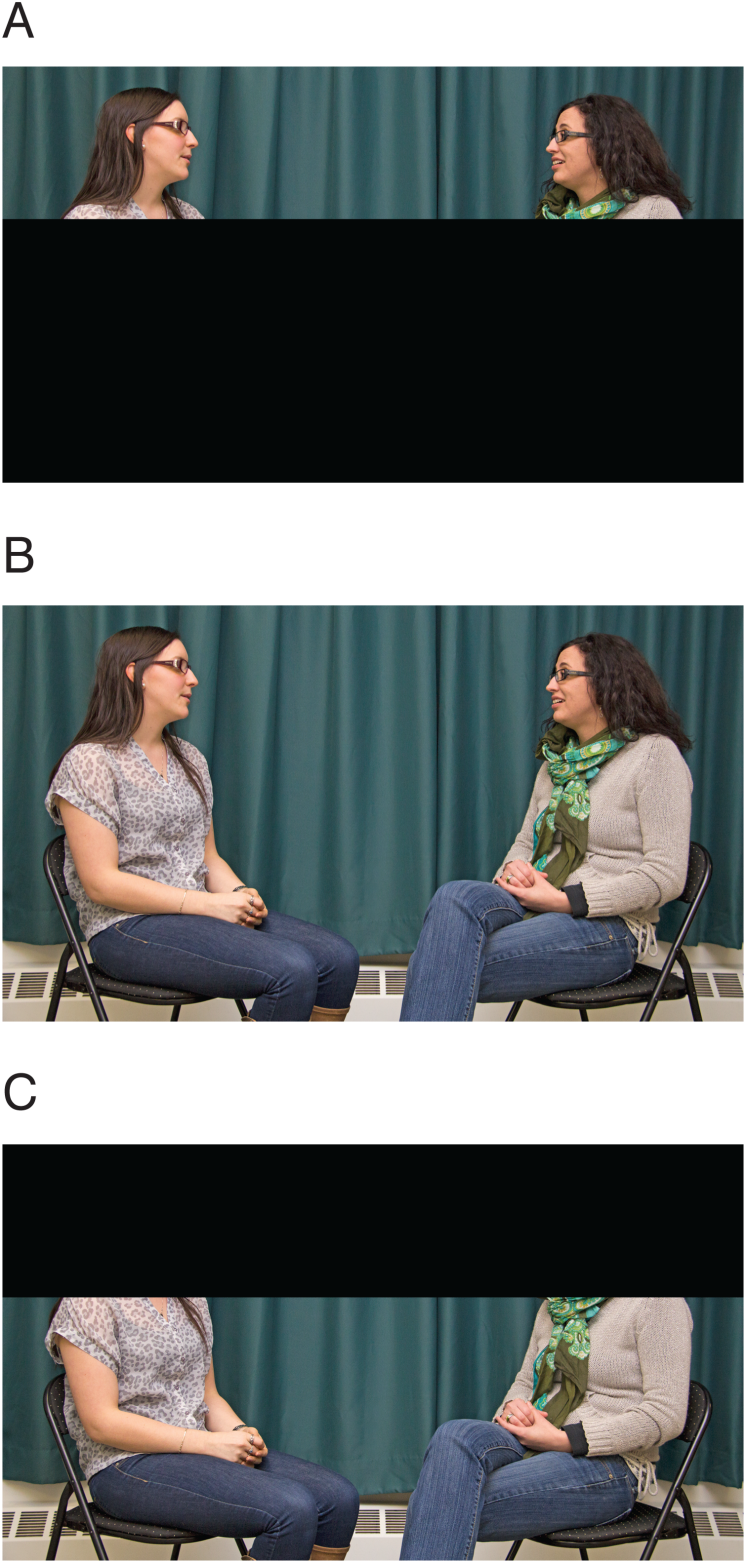
Example stimuli used in Experiment 2. A) Head-Only condition B) Head+Body condition C) Body-Only.

For Experiment 2, 24 clips (5 s long) were selected from the full 10-minute clips to include 12 same-gender friends and 12 same-gender stranger pairs. This set included six clips with the highest mean correlation and six clips with the lowest mean correlation within each affiliation category as determined by the analysis in Experiment 1. All videos were analyzed for the amount of motion where friends had a greater mean motion value (0.99 pixels/second, SE = 0.02) than strangers (0.89 pixels/second, SE = 0.016). Therefore, videos were controlled for the amount of motion so that all selected clips contained motion content that fell within half a standard deviation of the mean motion magnitude for both friends and strangers. Further, the clips were intentionally selected to be the highest and lowest correlation from each affiliation category. Although, on average, friends have a higher correlation than strangers, these clips were selected to distinguish high and low correlation perception irrespective of affiliation categorization. The average correlation for the “high correlation” and “low correlation” was equated. These clips were edited to create three types of stimuli which were presented in a between-subjects design: head-only (videos cropped at shoulders to show only head motion), head+body and body-only (videos cropped at shoulders to show only body motion (Figure 4 (The individual presented has given written informed consent as outlined in PLOS consent form to publish these case details)). This video cropping was based on post-experiment interviews with participants in a pilot experiment who reported focusing on face/head related information when making judgments. This is further supported by evidence from studies in social cognition that implicate the face region in cueing attention when observing social interaction [38].

Since all three viewing-information conditions were derived from the same clips, the average correlations for the high and low levels were not consistent across conditions (Table S1). Specifically, the head-only condition in Experiment 2 had a larger correlation difference between the high and low correlation levels than the other conditions. In part this is a function of scale where the full body videos are influenced by the correlations calculated for the overall motions. For the head motions, the correlations are calculated for smaller movements that will not influence the overall correlation greatly. Experiment 3 was conducted to confirm that the response pattern in the head+body information condition was not influenced by less extreme correlations. Using CMA, a new set of high and low coordination clips were selected from correlations computed for the whole body for this purpose.

#### Procedure

Twenty participants were assigned to one of three conditions in Experiment 2 (n = 60). A new set of 20 participants served in Experiment 3. Twenty four, five-second silent video clips were presented to each participant. Following each clip, participants were asked to identify the nature of the relationship between the interlocutors. Responses were given on a Likert scale from 1–7 where participants indicated whether interlocutors had just met (1) or were friends (7). Following the experiment, each participant was asked to record information they thought they used to make their judgments.

### Results

#### Experiment 2

**Figure 5:**
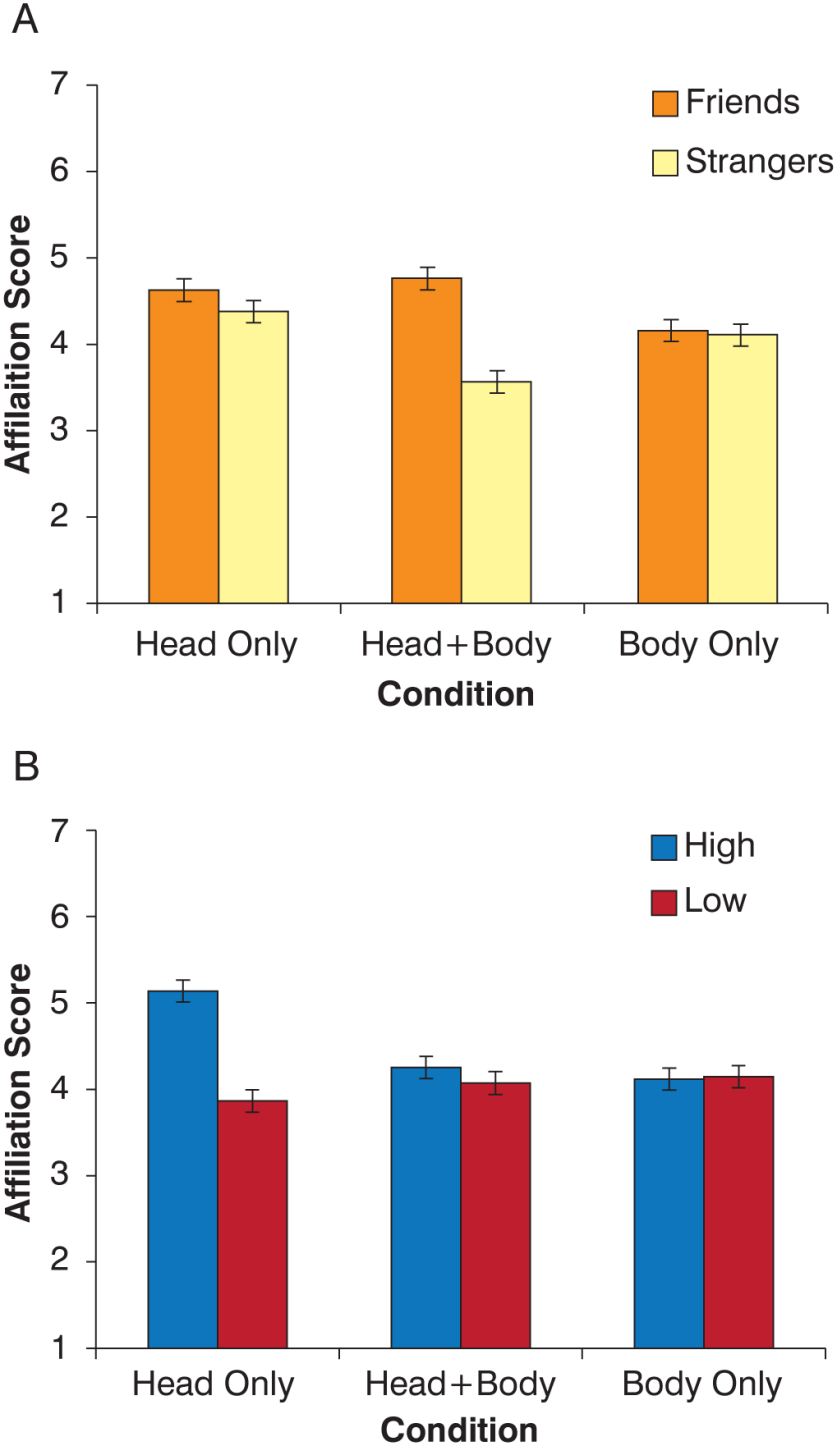
A) Mean affiliation score collapsed across correlation levels as a function of true affiliation for the three conditions. A greater score indicates a preference towards a judgment of ‘Friends’ and a lower score indicates preference towards a ‘strangers’ rating. When both head and body information was presented, participants could discriminate between friends and strangers accurately. B) Mean affiliation score collapsed across affiliation category as a function of correlation level for the three conditions. When only head information was presented, participants assumed a ‘friends’ affiliation for high correlation and a ‘strangers’ affiliation for low correlation, even if this may have been erroneous. Error Bars = Standard Error.

For Experiment 2, results of on analysis of variance (ANOVA) indicated a significant effect of condition (head-only vs. head+body vs. body-only; F(2,57) = 7.24, p<0.01). Participants were differentially influenced by the sources of information received, were able to discriminate between friends and strangers, and perceived highly correlated pairs as friends. However, the interpretation of these main effects is tempered by 2 interactions: the significant Viewing-Condition X Affiliation interaction (F (2, 57) = 15.81, p<0.01, partial eta squared = 0.36) and the significant Viewing-Condition X Correlation interaction (F (2, 57) = 7.74, p<0.01, partial eta squared = 0.21). There was no three-way interaction. The two-way interactions are presented in Figures 5a and 5b. Figure 5a shows that the interaction between condition and affiliation was driven by the head+body condition. When full information was provided to an observer, they accurately discriminated between friends and strangers. However, when information was restricted to only the head motion, observers strongly relied on correlation information (Figure 5b). These participants perceived high-coordination pairs as friends and low-coordination pairs as strangers regardless of the true affiliation between conversing individuals. Body-only information produced no systematic pattern in the observers’ affiliation ratings.

The results from two control experiments using separate sets of 15 participants (Control Experiment 1: head-only; Control Experiment 2: head+body) were consistent with our belief that movement was a strong contributing factor and that ratings were not significantly driven by static postural cues. These results are consistent with findings in face identification and audiovisual speech perception that indicate that dynamic information yields a richer set of information than static cues regarding identity and communication [39]–[41]. To test the relative salience of static and dynamic cues, we presented the first frame of every selected 5 s clip from the head-only and head+body conditions and asked participants to rate affiliation on the same 1–7 Likert scale. We note that testing static frames using a single first frame for each clip is very preliminary and other control strategies may be used to test the question of the contribution of motion. However, the appropriate control to use for comparing static and dynamic displays is not clear. Others have used randomly selected single frames [42], frame sequences that reduce the full frame rate [43] and frames showing extreme postures [44]. Ideally, showing all single frames from the clip individually would be the strongest test. Although testing all singly presented static frames from the videos would have provided the most ideal control, we suggest that the first frame would be representative of results for any randomly selected since the 5-second clips were selected from various points within the longer 10-minute videos. As expected, observers were not able to accurately discriminate between friends and strangers from static images. This suggests that static information may not be sufficient and that dynamic information is required to make quick social judgments. Previous studies have already demonstrated that the accuracy of social judgments such as emotion is significantly influenced by motion [45].

#### Experiment 3

**Figure 6:**
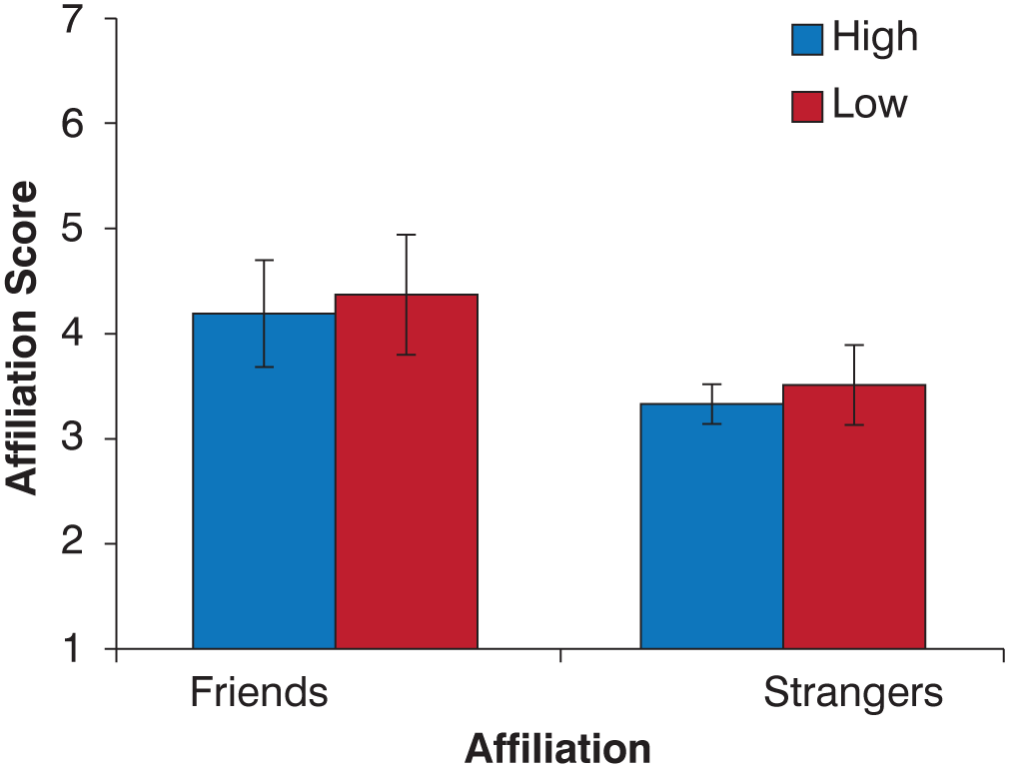
Mean affiliation score for full-body videos. Stimuli were calibrated to more clearly discriminate between high and low correlation levels. A greater score indicates a preference towards a judgment of ‘Friends’ and a lower score indicates preference towards a ‘strangers’ rating. Results replicate the results in the Head+Body condition in Experiment 2 where participants accurately discriminated between friends and strangers. Error Bars = Standard Error.

In Experiment 3, the new full-body stimuli replicated the full-body results from Experiment 2. Participants could accurately discriminate between friends and strangers (F(1,16) = 4.28, p = 0.05), however, coordination level did not affect the perceptual results (Figure 6). These results confirmed the perceptual results from Experiment 2 based on correlation values similar to the head-only stimuli used in that experiment.

### Discussion

Human conversation presents an example of joint behavior that relies on a coupled monitoring-guiding mechanism between interlocutors [9]. Movements during conversation may be guided by local perception/action systems in a manner similar to the collective behavior exhibited across many social animals (e.g. the synchronization and coordination of flocking birds and schooling fish [46]). Here, we provide quantitative evidence consistent with a coupled monitoring-guiding view of joint behavior during conversation [9] by showing that interacting individuals, especially friends, significantly coordinate their movements with each other. Moreover, this is a highly sensitive system and the higher correlations are observed at very small temporal offsets between talkers.

It is possible that differences in coordination are driven by factors other than affiliation. For example, it can be suggested that the overall amount of conversation may influence the coordination content during social interaction and hence observer judgments (e.g. friends may engage in a greater amount of conversation). However, this influence is unlikely as the conversation cannot occur without motion and the amount of motion resulting from conversation was tightly controlled. In addition, our measure of movement coordination, after selection of clips, is calculated based on the relative amount of motion within a signal. In our analyses, greater motion does not equate with more correlation; in fact, the greatest correlations can sometimes be computed from the smallest of motion magnitude values. Further, other influences may be considered in driving coordination; previous studies have suggested that shared knowledge between talkers increases behavioral coordination and may help with comprehension during interactions [47]–[48]. Similarly, familiarity with a particular topic has been shown to result in more coordinated and predictable turn-taking behavior [49]. We can speculate that friends may have greater common knowledge and more familiarity with that knowledge resulting in our observed correlation structure. However, previous studies looking at common ground have documented a gravitation towards shared information during conversation, even in individuals that have never met; individuals, in general, work towards finding shared interests to direct their conversations [50]–[52]. Future work examining cues such as shared knowledge and topic familiarity will help clarify the factors that influence conversational coordination.

When examining perceptual results in Experiments 2 and 3, we demonstrated that the availability of coordination cues is able to affect social decisions. When provided with full views of the talkers (head+body), observers replicated previously reported perceptual accuracy when judging affiliation [20]. However, this is not true when isolating parts of the same information. When viewing conversational movement restricted to the head region, observers are highly sensitive to the coordination at the expense of accuracy. Conversely, observers are unable to use body-only coordination. Coordination thus seems to be an informative cue that we are able to use when making judgments, but only when other cues are restricted. The results from the full-body condition indicate that conversational interaction provides a rich set of information (e.g. posture, facial expressions, proximity, gaze, etc.) in addition to movement correlation with which to make judgments.

At least with the single frames that we showed to subjects, static information was not sufficient to make judgments of affiliation. Previous studies have made a distinction between information retrieved from static versus dynamic displays. Although static displays are sufficient for inferring enduring qualities such as personality, this is not the case for conditions dependent on external factors such as the behavior of another person during social interaction; dynamic information is necessary for these judgments [53]. The importance of dynamic information has also been shown in visual speech perception [54], face perception [55] and emotional judgments [56]. Our data falls in line with previous studies that have shown that dynamic information is more perceptible to an observer and provides easier access to realistic representations of social information [57]. Humans are highly attuned to dynamic biological motion demonstrated by the robust responses in the action observations networks (premotor and motor areas) in the frontal cortex [58]. In additional, single-cell recordings in monkeys and neuroimaging studies in humans show that the superior temporal sulcus (STS) region is an important component of the perceptual system responsible for processing information required for the accurate analysis of the dispositions and intentions of others during social interaction; movements of the eyes, mouth, hands and body lead to activation in this region [59].

The fact that judgments made to head-only movement were influenced by coordination information is especially striking. This and observers’ inability to use body-only coordination suggests that conversational head motion resulting from speech might be a major contributor to coordination observed in previous studies (e.g. [3], [7]). This is not surprising since head motion is directly correlated with the auditory signal of speech [60] and can influence speech intelligibility [40]. Such acoustic-kinematic associations during speech may account for the fact that behavioral coordination is still observed when individuals interact without visual input from their partner [7]. Behavioral coordination thus may be a direct result of convergence of the speech signals of the individuals engaged in conversation. This is consistent with growing evidence that acoustic convergence contributes to communication and social bonding in primate and human populations. Vocal convergence has been observed among wild chimpanzees as they form social bonds. An examination of pant-hoot choruses in chimpanzees showed that an individual chimpanzee modified the acoustic properties of their pant-hoot to match those of the individual with whom chorusing [61]. Similarly, in human studies of speech and communication, phonetic convergence of properties such as pronunciation of use of lexical items of the course of conversation has been examined [6], [62]–[63]. This effect is especially pronounced when individuals are familiar with each other or share a common goal [14]; [64] indicating its role in social bonding and cooperation, much like non-human species [65].

We can draw two conclusions from these results: 1) Conversation evokes movement coordination and familiarity between interacting individuals enhances these movement patterns and 2) Judgment accuracy by external observers *can* be influenced by coordination information however, it is not the only cue informing our decisions. This demonstrates that human communication provides rich information for making judgments and many cues contribute to perceptual decisions. For example, previous work examining the components of conversation has implicated a higher instance of backchannelling behavior, less overlap between talkers and fewer interruptions in interactions between familiar individuals [66]. The contribution of such conversational cues as well as the manner in which multiple cues are integrated to produce our observed correlation structure and its perception is still unknown. Further work on the allocation of attention to these cues is required to fully understand the mechanism used to process coordination information.

In summary, quantifiable coordination differences during conversation based on affiliation were observed. When provided with restricted views of head motion, observers asked to discriminate between friends and strangers relied heavily on coordination information even though this was erroneous. This suggests that human social systems are attuned to coordination and this may facilitate communication formation of relationships. These findings have implications for gaining a clearer understanding of the common biological mechanism maintaining communication across many social animals.

## Supporting Information

Table S1 **Mean correlation values and standard error values for all experimental conditions.**
(DOC)
