# Correction: Movement Coordination during Conversation

**DOI:** 10.1371/journal.pone.0113316

**Published:** 2014-11-06

**Authors:** The PLOS ONE Staff

The third author’s name is spelled incorrectly. The correct name is Eric Vatikiotis-Bateson. The correct citation is: Latif N, Barbosa AV, Vatikiotis-Bateson E, Castelhano MS, Munhall KG (2014) Movement Coordination during Conversation. PLoS ONE 9(8): e105036. doi:10.1371/journal.pone.0105036

## References

[1] Latif N, Barbosa AV, Vatiokiotis-Bateson E, Castelhano MS, Munhall KG (2014) Movement Coordination during Conversation. PLoS ONE 9(8): e105036 doi:10.1371/journal.pone.0105036 25119189 10.1371/journal.pone.0105036 PMC4132081

