# Supplementary material for: Movement Coordination during Conversation
Source: PLoS One. 2014 Aug 13;9(8):e105036. doi: 10.1371/journal.pone.0105036 (PMC4132081; doi:10.1371/journal.pone.0105036)
Supplement: Table S1 — Mean correlation values and standard error values for all experimental conditions. (DOC) [file pone.0105036.s001.doc]

Supplementary Table

| **Head Only** | | | | |
| --- | --- | --- | --- | --- |
|  | **High Correlation** | | **Low Correlation** | |
| **Friends** | Mean=0.35;  SE=0.02 | | Mean=0.008;  SE=0.02 | |
| **Strangers** | Mean=0.34;  SE=0.01 | | Mean=0.004;  SE=0.0007 | |
| **Head+Body** | | | | |
|  | **High Correlation** | | **Low Correlation** | |
| **Friends** | Mean=0.47;  SE=0.001 | | Mean=0.24;  SE=0.02 | |
| **Strangers** | Mean=0.45;  SE=0.03 | | Mean=0.21;  SE=0.003 | |
| **Body+Only** | | | | |
|  | **High Correlation** | | **Low Correlation** | |
| **Friends** | Mean=0.58;  SE=0.001 | | Mean=0.47;  SE=0.04 | |
| **Strangers** | Mean=0.56;  SE=0.01 | | Mean=0.41;  SE=0.003 | |
| **Full Body (Experiment 3)** | | | | |
|  | | **High Correlation** | | **Low Correlation** |

| **Friends** | Mean=0.35;  SE=0.03 | Mean=0.10;  SE=0.002 |
| --- | --- | --- |
| **Strangers** | Mean=0.34;  SE=0.01 | Mean=0.08;  SE=0.007 |
